# Associations of Hysterectomy and Oophorectomy with Dementia Risk: A Systematic Review and Meta-Analysis of Observational Studies

**DOI:** 10.3390/jcm15187247

**Published:** 2026-09-17

**Authors:** Xiaotong Fu, Shuiqing Xu, Yang Wang, Ming Wang

**Affiliations:** 1 Department of Gynecologic Oncology, Beijing Obstetrics and Gynecology Hospital, Capital Medical University, Beijing Maternal and Child Health Care Hospital, 17 Qihelou St, Dongcheng District, Beijing 100006, China; fuxiaotong98@163.com (X.F.); xsq@mail.ccmu.edu.cn (S.X.); 2 Department of Neurosurgery, Beijing Chao-Yang Hospital, Capital Medical University, Beijing 100020, China; 3 Department of Obstetrics and Gynecology, Beijing Chao-Yang Hospital, Capital Medical University, Beijing 100020, China; 4 Laboratory for Clinical Medicine, Capital Medical University, Beijing 100069, China

**Keywords:** hysterectomy, oophorectomy, dementia, Alzheimer’s disease, mild cognitive impairment, meta-analysis

## Abstract

**Objectives:** The aim was to evaluate associations of hysterectomy and oophorectomy with dementia-related outcomes while distinguishing specific surgical procedures. **Methods:** PubMed, Embase, and the Cochrane Library were searched from inception and updated through 20 August 2026. Adjusted hazard ratios (HRs), risk ratios (RRs), and odds ratios (ORs) were synthesized on the log scale using generic inverse-variance random-effects models with restricted maximum likelihood. Quantitative pools were restricted to explicitly defined procedures; mild cognitive impairment (MCI) was kept separate from dementia and Alzheimer’s disease (AD). **Results:** Nine reports involving 2,693,443 participants were included in the systematic review, of which five contributed to at least one procedure-specific meta-analysis. Hysterectomy without documented adnexal surgery was not clearly associated with dementia/AD (4 studies; pooled ratio 0.94, 95% confidence interval [CI] 0.84–1.05; I^2^ = 82.2%). Hysterectomy with bilateral salpingo-oophorectomy/oophorectomy also showed no clear association (2 studies; 1.04, 95% CI 0.67–1.62; I^2^ = 83.6%), nor did bilateral oophorectomy alone (4 studies; 0.99, 95% CI 0.88–1.12; I^2^ = 75.6%). No independently extractable study evaluated hysterectomy with bilateral salpingectomy. **Conclusions:** Procedure-specific analyses did not demonstrate a clear association with dementia or AD, but substantial heterogeneity, residual confounding, and exposure misclassification limit certainty and preclude causal interpretation.

## 1. Introduction

Dementia is a clinical syndrome characterized by progressive cognitive decline, resulting in impaired occupational performance, learning capacity, activities of daily living, and social functioning. Affected individuals typically exhibit varying degrees of decline in memory, language ability, and executive function [1]. Alzheimer’s disease (AD) is the most common cause of dementia [2], accounting for approximately 60% to 80% of cases [3].

Dementia is a complex disorder influenced by genetic and environmental factors. The apolipoprotein E (APOE) ε4 allele is an important genetic susceptibility factor and has been associated with differences in hippocampal and amygdala volumes [4]. Modifiable factors, including low educational attainment, smoking, obesity, sensory impairment, hypertension, diabetes, excessive alcohol consumption, physical inactivity, head trauma, depression, social isolation, air pollution, and high low-density lipoprotein (LDL) cholesterol, may also contribute to dementia risk [5]. Experimental work has shown that a high-fat diet can reduce hippocampal ApoE protein levels in an isoform-dependent manner [6]. Greater adherence to Mediterranean-style dietary patterns has generally been associated with lower risks of dementia and AD, although the magnitude of the association varies among studies [7]. Active cigarette smoking has been associated with elevated cerebrospinal-fluid oxidative stress biomarkers in cognitively normal older adults and patients with probable AD [8]. In aged mice, treadmill exercise improved cognition and reduced glial activation while increasing fibronectin type III domain-containing protein 5 (FNDC5)/irisin, sirtuin 1 (SIRT1), brain-derived neurotrophic factor (BDNF), and antioxidant-pathway markers [9].

Estrogen loss is associated with immediate menopausal symptoms and may also influence longer-term brain health. Women account for a larger proportion of people living with dementia, although longevity, selective survival, and possible sex-specific biological and social mechanisms complicate interpretation of this difference [10,11]. Estrogen may exert neurotrophic and neuroprotective effects through several pathways [12,13,14]. Therefore, surgical procedures that alter ovarian hormone exposure, particularly bilateral oophorectomy, have been investigated as possible risk factors for dementia [15]. Hysterectomy and oophorectomy are common gynecological procedures, but their endocrine consequences differ substantially depending on whether the ovaries are conserved.

In the REDLINC XII study of 1185 postmenopausal women, surgical menopause was associated with a higher prevalence of mild cognitive impairment (MCI) than spontaneous menopause (odds ratio [OR] = 1.47, 95% CI = 1.01–2.16) [16]. A UK Biobank neuroimaging study of 19,846 women found that current menopausal hormone therapy (MHT) users had an older estimated brain age by up to 9 months and smaller hippocampal volumes than never-users; among MHT users, those with a history of hysterectomy and/or bilateral oophorectomy had a younger gray-matter brain age than users without such surgery [17]. A previous systematic review found limited evidence overall but suggested that surgical menopause induced by bilateral oophorectomy at age 45 years or younger may be associated with a higher risk of dementia or cognitive decline [18]. Given these inconsistent findings, this meta-analysis examined population-based evidence on hysterectomy, oophorectomy, dementia, AD, and MCI while distinguishing surgical procedures wherever possible.

## 2. Materials and Methods

This meta-analysis was conducted following the guidelines of the Preferred Reporting Items for Systematic Reviews and Meta-Analyses (PRISMA) [19]. The protocol was pre-registered on the International Prospective Register of Systematic Reviews (PROSPERO) platform (registration number: CRD42024585062).

### 2.1. Data Sources and Searches

PubMed, the Cochrane Library, and Embase were searched from database inception and updated through 20 August 2026. Controlled vocabulary and free-text terms were used for hysterectomy, salpingectomy, oophorectomy/ovariectomy, dementia, Alzheimer’s disease, and cognitive impairment. Reference lists of eligible articles and relevant reviews were also checked. All three database searches were completed and incorporated into the updated screening process.

### 2.2. Eligibility Criteria

Eligible studies were observational cohort, case–control, or cross-sectional studies that: (1) compared women with defined hysterectomy and/or oophorectomy exposure with an appropriate non-exposed group; (2) reported dementia, Alzheimer’s disease, vascular dementia, or MCI; and (3) provided an adjusted hazard ratio (HR), risk ratio (RR), or OR with a 95% confidence interval (CI), or sufficient information to derive it. Cross-sectional evidence was retained only for the separate MCI synthesis and was not combined with dementia/AD. For the procedure-specific analyses, procedures were classified as hysterectomy without documented adnexal surgery, hysterectomy with bilateral salpingectomy, hysterectomy with bilateral salpingo-oophorectomy/oophorectomy, unilateral oophorectomy alone, or bilateral oophorectomy alone. Estimates with unclear laterality or unspecified concomitant adnexal surgery were excluded from strict procedure-specific pooling and retained for narrative synthesis.

### 2.3. Study Selection

Two reviewers (XTF and SQX) independently screened the literature for eligibility and exclusion criteria. Initially, they excluded duplicate and irrelevant articles based on titles and abstracts. The full texts of potentially eligible articles were then reviewed to confirm eligibility. Discrepancies were resolved by a third reviewer (MW), who served as an arbiter.

### 2.4. Data Extraction

Two reviewers (XTF and SQX) independently extracted study design, population, sample size, exposure definition (including laterality and concomitant salpingectomy/oophorectomy), comparator, outcome definition, adjusted covariates, and maximally adjusted ratio estimates with 95% CIs using predesigned forms and established data-extraction principles [20]. Disagreements were resolved by discussion with a third reviewer. Multiple publications from the same underlying cohort were checked to avoid double counting; the most relevant non-overlapping estimate was retained for each analysis.

### 2.5. Risk of Bias Assessment

Risk of bias was assessed independently by two reviewers. The Newcastle–Ottawa Scale (NOS) was used for cohort and case–control studies [21], whereas the 20-item Appraisal Tool for Cross-Sectional Studies (AXIS) was used for the single cross-sectional study [22]. Disagreements were resolved by consensus. Because total scores can obscure domain-specific limitations, confounding control, exposure ascertainment, outcome ascertainment, selection, and study-design limitations were considered when interpreting the evidence. A sensitivity analysis excluding cohort or case–control studies with NOS scores below 7 was conducted; it removed no study because all such studies scored 8 or 9. The cross-sectional MCI study was retained only narratively and was not included in dementia/AD pooling.

### 2.6. Statistical Analysis

Meta-analyses were performed using Review Manager (RevMan) version 5.4 (The Cochrane Collaboration, London, UK) and R software version 4.3.0 (R Foundation for Statistical Computing, Vienna, Austria).Adjusted HRs, RRs, and ORs were analyzed on the natural-log scale as ratio measures using generic inverse-variance methods. Random-effects models were fitted using restricted maximum likelihood (REML), irrespective of the heterogeneity test result. Statistical heterogeneity was described using Cochran’s Q, I^2^, and τ^2^. Leave-one-out analyses were performed for groups containing at least three studies. Because AD is a major cause of dementia but is not identical to all-cause dementia, analyses combining these outcomes were considered exploratory. Procedure-specific models were conducted only when at least two independent studies provided explicitly classifiable estimates. A formal between-subgroup test was not performed because some studies contributed correlated estimates to more than one exposure category and within-study covariance was unavailable. Funnel plots and Egger tests were not used because every quantitative comparison contained fewer than 10 studies. Two-sided *p* < 0.05 was considered statistically significant for descriptive tests.

## 3. Results

### 3.1. Literature Search

**Figure 1:**
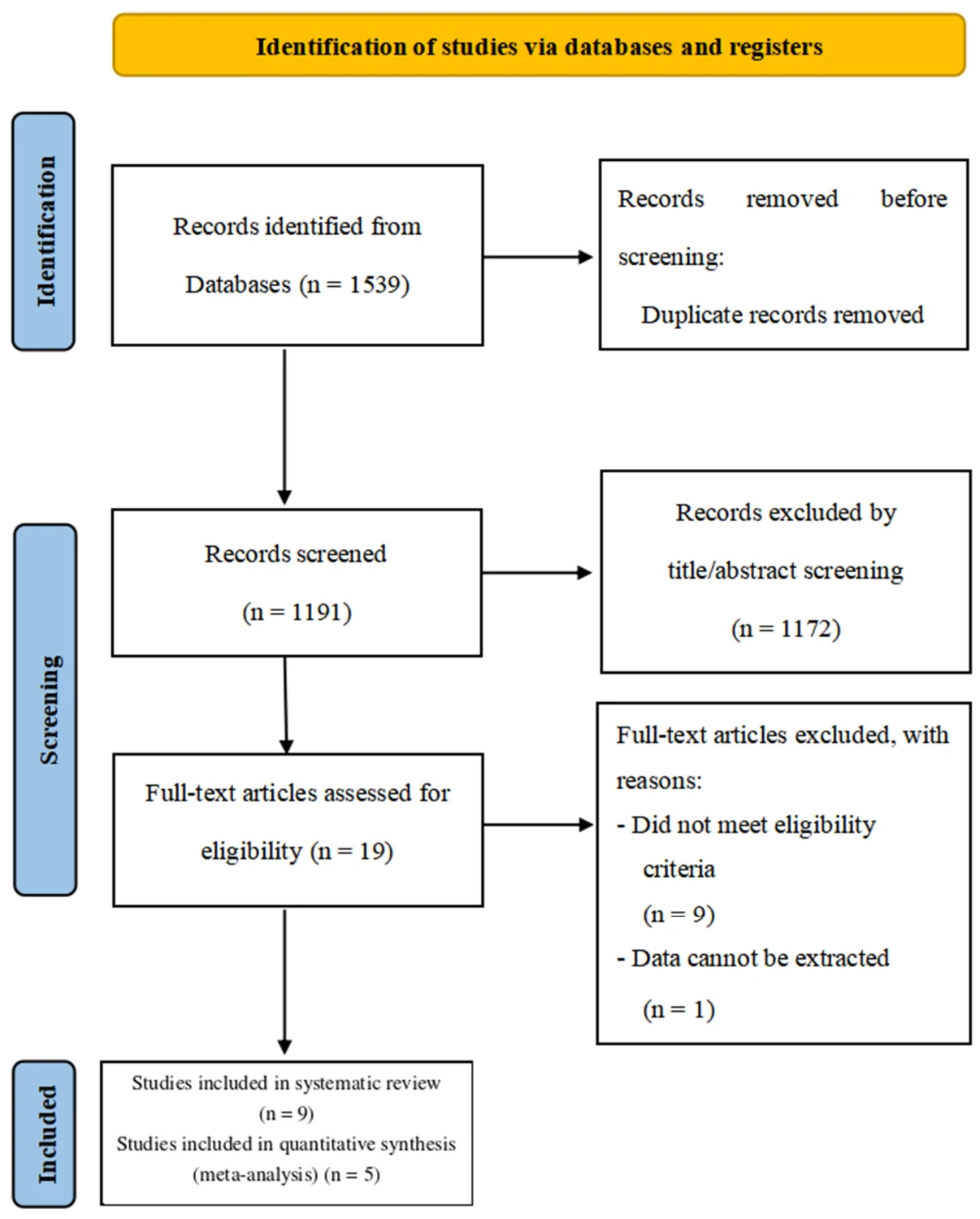
Flow chart of literature screening.

The original search identified 1446 records; after removal of 348 duplicates, 1098 records were screened, 9 full-text reports were assessed, and 6 studies were included. The updated PubMed search identified 93 records. The updated Embase and Cochrane Library searches identified no additional eligible studies. After title and abstract screening, 10 reports underwent full-text assessment. Six did not meet the eligibility criteria, one lacked sufficient full-text or extractable procedure-specific data, and three new reports were included. The updated systematic review therefore comprised 9 eligible studies/reports involving 2,693,443 participants, of which 5 provided independently extractable estimates for at least one predefined procedure-specific quantitative analysis (Figure 1).

### 3.2. Study Characteristics

The updated evidence base comprised observational cohort, case–control, and cross-sectional studies published from 2007 to 2026 [23,24,25,26,27,28,29,30,31] (Supplementary Table S1). The newly included reports were Peterson et al. (2025), Yoon and Yuk (2026), and Li et al. (2026) [29,30,31]. Their surgical definitions differed materially: Peterson et al. [29] did not report whether oophorectomy accompanied hysterectomy; Yoon and Yuk [30] distinguished hysterectomy without versus with adnexal surgery but did not separate salpingectomy from oophorectomy in the combined category; and Li et al. [31] reported menopause due to hysterectomy or ovariectomy without sufficient concomitant-procedure or laterality detail for strict pooling.

### 3.3. Quality Assessment

All cohort and case–control studies received NOS scores of 8 or 9, indicating generally adequate selection and outcome ascertainment; the cross-sectional MCI study was assessed separately using AXIS. However, residual confounding and variation in surgical coding remained important domain-specific limitations. Excluding studies with NOS scores below 7 did not change the analysis because no study met that exclusion criterion (Supplementary Table S2).

### 3.4. Procedure-Specific Associations with Dementia or Alzheimer’s Disease

**Figure 2:**
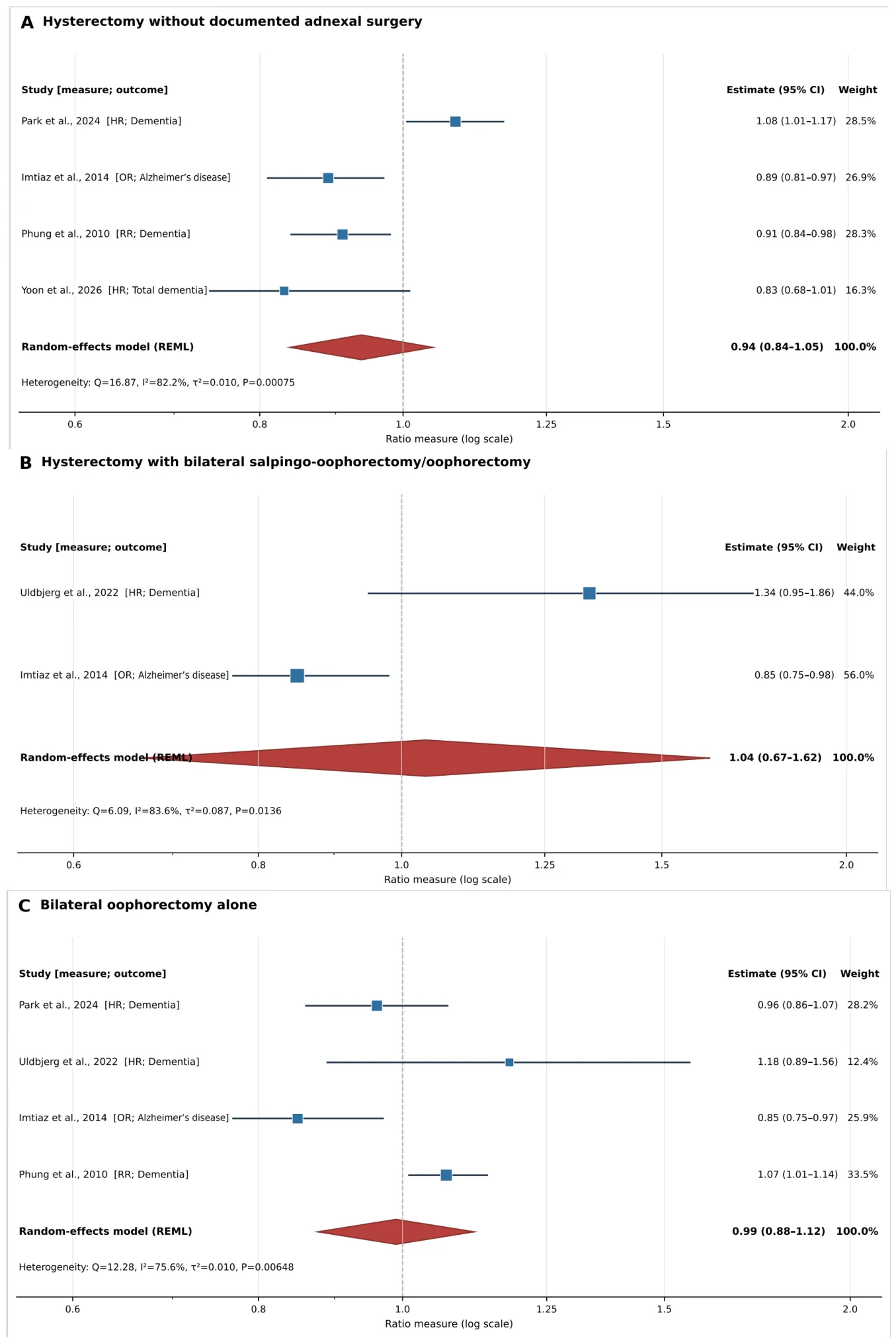
Procedure-specific forest plots of the associations between gynecological surgery and dementia or Alzheimer’s disease, based on five studies that provided independently extractable estimates for the predefined surgical categories. (**A**) Hysterectomy without documented adnexal surgery; (**B**) hysterectomy with bilateral salpingo-oophorectomy/oophorectomy; and (**C**) bilateral oophorectomy alone. Study-specific adjusted effect estimates and pooled ratios are presented with 95% confidence intervals. Pooled estimates were calculated using random-effects models. Some studies contributed estimates to more than one procedure-specific analysis. The vertical line at 1.0 indicates no association. CI, confidence interval [23,24,26,27,30].

Four studies provided classifiable estimates for hysterectomy without documented adnexal surgery [23,26,27,30]. The pooled ratio was 0.94 (95% CI 0.84–1.05), with substantial heterogeneity (Q = 16.87, df = 3, *p* < 0.001; I^2^ = 82.2%; τ^2^ = 0.010). Two studies evaluated hysterectomy with bilateral salpingo-oophorectomy/oophorectomy [24,26]; the pooled ratio was 1.04 (95% CI 0.67–1.62; Q = 6.09, df = 1, *p* = 0.014; I^2^ = 83.6%; τ^2^ = 0.087). Four studies evaluated bilateral oophorectomy alone [23,24,26,27]; the pooled ratio was 0.99 (95% CI 0.88–1.12; Q = 12.28, df = 3, *p* = 0.006; I^2^ = 75.6%; τ^2^ = 0.010). None showed a clear overall association with dementia or AD. No independently extractable estimate was available for hysterectomy with bilateral salpingectomy or for unilateral oophorectomy alone (Figure 2).

### 3.5. Evidence Not Eligible for Strict Procedure-Specific Pooling

Peterson et al. reported an adjusted HR of 1.08 (95% CI 0.94–1.26) for AD dementia after premenopausal hysterectomy, but ovarian surgery status at hysterectomy was unavailable [29]. Yoon and Yuk reported an HR of 1.06 (95% CI 0.69–1.63) for total dementia after hysterectomy with unspecified adnexal surgery [30]. Li et al. reported an RR of 1.02 (95% CI 0.56–1.84) after menopause due to hysterectomy and 2.17 (95% CI 1.16–4.08) after ovariectomy, but concomitant procedures and ovariectomy laterality were insufficiently specified [31]. These findings were retained narratively, and the single MCI estimate (OR 1.56, 95% CI 1.09–2.24) was kept separate from dementia/AD [25]. In leave-one-out analyses, estimates ranged from 0.90 to 0.96 for hysterectomy without documented adnexal surgery and from 0.95 to 1.04 for bilateral oophorectomy alone. Omission of Park et al. [23] changed the former estimate to 0.90 (95% CI 0.85–0.95), indicating sensitivity to individual studies. Leave-one-out analysis was not informative for the two-study hysterectomy-with-oophorectomy group.

### 3.6. Publication Bias

Publication bias was not formally assessed because each quantitative comparison included fewer than 10 studies, for which funnel-plot asymmetry and regression tests are unreliable.

## 4. Discussion

### 4.1. Main Findings

This updated review identified nine eligible reports and replaced the previous broad pooling of heterogeneous surgical exposures with procedure-specific analyses. No clear association with dementia or AD was observed for hysterectomy without documented adnexal surgery, hysterectomy with bilateral salpingo-oophorectomy/oophorectomy, or bilateral oophorectomy alone. However, all pooled analyses showed substantial heterogeneity and several newly identified estimates could not be assigned to a strict surgical category. The absence of a pooled association should therefore not be interpreted as proof of no effect for a specific age, indication, or surgical context.

### 4.2. Interpretation of Findings

Separating procedures materially changed the interpretation. Hysterectomy with ovarian conservation does not cause the immediate hormone loss produced by bilateral oophorectomy, but it may still affect ovarian function. A prospective cohort found that women undergoing hysterectomy with ovarian preservation had a higher risk of subsequent ovarian failure than women with intact uteri, although the study could not fully separate a surgical effect from the underlying indication [32]. Disruption of the uterine–ovarian blood supply and accelerated follicular depletion have been proposed as possible explanations, but these mechanisms remain uncertain. The indication for hysterectomy may also be relevant because fibroids, endometriosis, abnormal bleeding, inflammation, vascular risk, and metabolic factors could influence later cognitive outcomes independently of surgery. These mechanisms are difficult to separate from confounding in observational studies. Reviews of salpingo-oophorectomy performed with benign hysterectomy likewise emphasize ovarian conservation and precise procedure definitions [33]. Differences in age at surgery, menopausal status, indication, MHT, follow-up, and outcome ascertainment plausibly contributed to the high I^2^ values. HRs, RRs, and ORs were combined only as exploratory ratio measures because they are not identical estimands.

Biological hypotheses involving abrupt estrogen loss, neurovascular changes, inflammation, and amyloid-related pathways remain plausible, particularly after premenopausal bilateral oophorectomy. Recent observational and imaging studies have described AD-related risk or resilience factors and imaging biomarker differences among women with early bilateral oophorectomy [34,35]. Experimental and translational studies also support estrogen-related neuroprotective and mitochondrial pathways [36,37]. Other work has examined associations of lifetime estrogen exposure or ovarian removal with domain-specific cognition, sleep physiology, brain structure, and menopausal hormone therapy [38,39,40,41]. Nevertheless, the observational evidence cannot separate the effect of surgery from indication, baseline vascular and metabolic risk, genetic susceptibility, socioeconomic factors, or subsequent hormone therapy. The estimates should therefore be interpreted as associations rather than causal effects.

The newly included studies reinforce the need for precise exposure definitions. Peterson et al. could not determine ovarian status at hysterectomy [29]; Yoon and Yuk grouped different adnexal operations together [30]; and Li et al. did not provide sufficient laterality or concomitant-procedure information for strict pooling [31]. These are not minor reporting details: they determine whether a study addresses uterine removal, loss of ovarian function, opportunistic salpingectomy, or a combination of procedures.

### 4.3. Implications and Limitations

Current evidence does not support using a single pooled estimate for ‘hysterectomy or oophorectomy’ in clinical counseling. Decisions should remain individualized according to surgical indication, age, ovarian conservation, baseline dementia risk, and the expected benefits and harms of each procedure. Major limitations include the small numbers of studies within each strict subgroup, substantial heterogeneity, residual confounding, possible exposure misclassification, mixed effect measures, exploratory pooling of AD with broader dementia outcomes, incomplete reporting of laterality and concomitant procedures, and the inability to test differences between correlated subgroups. The bilateral salpingectomy category remains an important evidence gap. The searches of PubMed, Embase, and the Cochrane Library were updated through 20 August 2026. Future prospective studies should use standardized exposure and dementia definitions; report hysterectomy alone, salpingectomy, and unilateral and bilateral oophorectomy separately; adjust for indication and other confounders; and provide sufficiently long follow-up.

## 5. Conclusions

In procedure-specific random-effects analyses, hysterectomy without documented adnexal surgery, hysterectomy with bilateral salpingo-oophorectomy/oophorectomy, and bilateral oophorectomy alone were not clearly associated with dementia or Alzheimer’s disease. Confidence intervals, substantial heterogeneity, residual confounding, and incomplete surgical classification limit certainty. No conclusion could be drawn for hysterectomy with bilateral salpingectomy. Better-defined prospective studies are required before causal or procedure-specific clinical inferences can be made.

## Data Availability

All data used in this meta-analysis were extracted from published studies and are presented in the article and its Supplementary Materials. No new participant-level dataset was generated.

## Supplementary Materials

The following supporting information can be downloaded at https://www.mdpi.com/article/10.3390/jcm15187247/s1, Supplementary Table S1: Characteristics of the included studies; Supplementary Table S2: Design-appropriate quality assessment of the included studies.

## Abbreviations

The following abbreviations are used in this manuscript:

AD	Alzheimer’s disease
MCI	Mild cognitive impairment
NOS	Newcastle–Ottawa Scale
PROSPERO	International Prospective Register of Systematic Reviews
BSO	Bilateral salpingo-oophorectomy
MHT	Menopausal hormone therapy
HT	Hormone therapy
MoCA	Montreal Cognitive Assessment
BDNF	Brain-derived neurotrophic factor
APOE	Apolipoprotein E
BMI	Body mass index
OVX	Ovariectomized
HR	Hazard ratio
RR	Risk ratio
OR	Odds ratio
CI	Confidence interval
REML	Restricted maximum likelihood
AXIS	Appraisal Tool for Cross-Sectional Studies
FNDC5	Fibronectin type III domain-containing protein 5
SIRT1	Sirtuin 1
LDL	Low-density lipoprotein
