# Supplementary material for: Associations of Hysterectomy and Oophorectomy with Dementia Risk: A Systematic Review and Meta-Analysis of Observational Studies
Source: J Clin Med. 2026 Sep 17;15(18):7247. doi: 10.3390/jcm15187247 (PMC13607537; doi:10.3390/jcm15187247)
Supplement: Supplementary file 1 [file jcm-15-07247-s001.zip › Supplementary Table S1 Study Characteristics.pdf]

Supplementary Table S1. Characteristics of the included studies

Author	Year	Country	Study type	Sample size	Outcome ascertainment	Exposure / comparator	Age	Follow-up	Main adjustment
Park et al.	2024	UK	Prospective cohort	273,260	Linked diagnosis codes	Hysterectomy; bilateral oophorectomy / no surgery	56.3±8.0	Prospective UK Biobank registry follow-up; duration not stated in the abstract	Age, education, APOE, family history, vascular/lifestyle factors
Uldbjerg et al.	2022	Denmark	Prospective cohort	24,851	National registers	Oophorectomy categories / no oophorectomy	Register cohort	334,420 person-years; followed to 31 December 2018	Age, calendar time, parity, education, comorbidity
Blümel et al.	2022	Latin America	Cross-sectional study	941	MoCA-defined MCI	Bilateral oophorectomy / no bilateral oophorectomy	Postmenopausal	Cross-sectional; not applicable	Age, education, comorbidity, hormone therapy
Imtiaz et al.	2014	Finland	Nationwide case-control	38,086 (19,043 cases; 19,043 controls)	Register-defined AD	Hysterectomy and/or oophorectomy / no surgery	Women with/without AD	Case-control; not applicable	Age and registered clinical/socioeconomic covariates
Phung et al.	2010	Denmark	Historical cohort	2,313,388	National dementia registers	Hysterectomy and oophorectomy categories / no surgery	Adult women	From 1977/age 40 to dementia, death, emigration, or 31 December 2006	Age, calendar period, and register covariates
Rocca et al.	2007	USA	Population cohort	2,961 (1,489 exposed; 1,472 referents)	Clinical cognitive impairment/dementia	Premenopausal oophorectomy / referents	Premenopausal surgery	Followed through death or end of study	Education and clinical covariates
Peterson et al.	2025	USA	Prospective cohorts	2,862	Clinical AD diagnosis	Premenopausal hysterectomy / no hysterectomy	77.1±7.7 at baseline	Mean 8.6 ± 5.6 years	Race, early-life socioeconomic status, education; age time scale
Yoon & Yuk	2026	Korea	Nationwide retrospective cohort	33,636 (16,818 per matched group)	ICD-10 claims for dementia	Hysterectomy without/with adnexal surgery / matched controls	40–59 at surgery	Median 11.4 years	Propensity-score matched demographic and clinical variables
Li et al.	2026	China	Prospective cohort	5,606	Clinical AD diagnosis	Menopause due to hysterectomy or ovariectomy / natural menopause	65.7±6.5	Mean 3.96 years; maximum 7 years	Age, race, education, socioeconomic, BMI, vascular/lifestyle factors, depression

Abbreviations: AD, Alzheimer disease; APOE, apolipoprotein E; BMI, body mass index; MCI, mild cognitive impairment; MoCA, Montreal Cognitive Assessment.
