# Supplementary material for: Associations of Hysterectomy and Oophorectomy with Dementia Risk: A Systematic Review and Meta-Analysis of Observational Studies
Source: J Clin Med. 2026 Sep 17;15(18):7247. doi: 10.3390/jcm15187247 (PMC13607537; doi:10.3390/jcm15187247)
Supplement: Supplementary file 1 [file jcm-15-07247-s001.zip › Supplementary Table S2 Quality Assessment.pdf]

Supplementary Table S2. Design-appropriate quality assessment of the included studies

Panel A. Summary of quality-assessment methods and results

Study	Design	Assessment tool	Result	Main limitations considered
Park et al., 2024	Prospective cohort	NOS—cohort version	9/9	Residual confounding; surgical coding
Uldbjerg et al., 2022	Prospective cohort	NOS—cohort version	8/9	Register-based exposure and outcome definitions
Blümel et al., 2022	Cross-sectional	AXIS	10 Yes; 10 Unclear	Cross-sectional temporality; self-reported surgical history; residual confounding
Imtiaz et al., 2014	Case-control	NOS—case-control version	8/9	Retrospective register exposure assessment
Phung et al., 2010	Historical cohort	NOS—cohort version	9/9	Register coding and residual confounding
Rocca et al., 2007	Population cohort	NOS—cohort version	8/9	Surgical indication and residual confounding
Peterson et al., 2025	Prospective cohorts	NOS—cohort version	8/9	Volunteer cohorts; self-reported exposure; ovarian status at hysterectomy unavailable
Yoon & Yuk, 2026	Retrospective cohort	NOS—cohort version	9/9	Residual confounding; adnexal procedures incompletely separated
Li et al., 2026	Prospective cohort	NOS—cohort version	8/9	Outcome-assessor blinding not reported; self-reported exposure; laterality unclear

Panel B. Newcastle–Ottawa Scale assessment of cohort studies

Study	Selection (maximum 4 stars)	Comparability (maximum 2 stars)	Outcome (maximum 3 stars)	Total
Park et al., 2024	★★★★	★★	★★★	9/9
Uldbjerg et al., 2022	★★★	★★	★★★	8/9
Phung et al., 2010	★★★★	★★	★★★	9/9
Rocca et al., 2007	★★★★	★★	★★	8/9
Peterson et al., 2025	★★★★	★★	★★	8/9
Yoon & Yuk, 2026	★★★★	★★	★★★	9/9
Li et al., 2026	★★★★	★★	★★	8/9

Panel C. Newcastle–Ottawa Scale assessment of the case-control study

Study	Selection (maximum 4 stars)	Comparability (maximum 2 stars)	Exposure (maximum 3 stars)	Total
Imtiaz et al., 2014	★★★★	★★	★★	8/9

NOS note: The cohort and case-control versions of the NOS contain design-specific domains. Domain-level judgments are therefore presented in separate panels. Higher star counts indicate that more NOS criteria were met; domain-specific limitations were considered alongside totals.

Panel D. Item-level AXIS appraisal of the cross-sectional REDLINC X study

No.	AXIS appraisal item	Judgment	Evidence/rationale
1	Were the aims/objectives of the study clear?	Yes	The objective was explicitly stated.
2	Was the study design appropriate for the stated aim(s)?	Yes	The analytical cross-sectional design was suitable for estimating an association, but not causality.
3	Was the sample size justified?	Unclear	No a priori sample-size or power calculation was identified.
4	Was the target/reference population clearly defined?	Yes	Postmenopausal women aged ≥60 years from six Latin American countries were defined.
5	Was the sample frame taken from an appropriate population base?	Unclear	The sampling frame and its coverage of the wider target population were insufficiently described.
6	Was the selection process likely to select representative participants?	Unclear	Recruitment and representativeness were insufficiently reported.
7	Were measures undertaken to address and categorise non-responders?	Unclear	No procedures for non-responders were clearly reported.
8	Were the risk factor and outcome variables appropriate to the aims?	Yes	Bilateral oophorectomy/MHT history and MoCA-defined MCI were relevant variables.
9	Were variables measured correctly using previously trialled, piloted, or published instruments?	Unclear	MoCA is published, but validation of the reported surgical and hormone-therapy histories was unclear.
10	Was statistical significance and/or precision clearly defined?	Yes	Odds ratios, 95% confidence intervals, and P values were reported.
11	Were the methods sufficiently described to enable repetition?	Yes	The outcome definition and multivariable logistic-regression approach were described.
12	Were the basic data adequately described?	Yes	Key participant and exposure characteristics were reported.
13	Does the response rate raise concerns about non-response bias?	Unclear	A sufficiently detailed response rate or eligible-to-analysed flow was not reported.
14	If appropriate, was information about non-responders described?	Unclear	Characteristics of non-responders were not reported.
15	Were the results internally consistent?	Yes	Descriptive results, adjusted estimates, and conclusions were internally coherent.
16	Were results presented for all analyses described in the methods?	Unclear	Complete concordance with all prespecified analyses could not be confirmed.
17	Were the discussion and conclusions justified by the results?	Yes	The reported association was supported, provided it was not interpreted causally.
18	Were the limitations of the study discussed?	Unclear	The extent of discussion of sampling, self-report, confounding, and temporality was not fully clear.
19	Were funding sources or conflicts of interest reported and unlikely to invalidate interpretation?	Yes	Disclosure information was provided, with no conflict identified that clearly invalidated interpretation.
20	Was ethical approval or participant consent attained?	Unclear	Ethics approval and informed-consent procedures were not confirmed in the information available for appraisal.

AXIS note: The AXIS developers do not recommend converting the 20 items into a numerical cutoff score. Overall appraisal: moderate risk of bias/some concerns, primarily because of uncertain sampling representativeness, non-response reporting, exposure ascertainment, residual confounding, and cross-sectional temporality.

Study appraised with AXIS: Blümel JE et al. Association of bilateral oophorectomy and menopause hormone therapy with mild cognitive impairment: the REDLINC X study. Climacteric. 2022;25(2):195–202. doi:10.1080/13697137.2021.1951203.
